# Determination of Heavy Metals in *Alpinia oxyphylla* Miq. Collected from Different Cultivation Regions

**DOI:** 10.1155/2016/7571802

**Published:** 2016-05-16

**Authors:** Dan Zhou, Yurong Fu, Weiyong Lai, Junqing Zhang

**Affiliations:** ^1^School of Pharmacy, Hainan Medical University, Haikou 571199, China; ^2^School of Pharmacy, Hainan Medical University, Hainan Provincial Key Laboratory of R&D of Tropical Herbs, Haikou 571199, China

## Abstract

20 batches of* Alpinia oxyphylla* Miq. were collected from Yunnan, Guangdong, Guangxi, and Hainan province in China. The contents of heavy metals of As, Hg, Pb, Cd, and Cu were determined and compared. The results indicated that geographical source might be a major factor to influence the contents of heavy metals of arsenic (As), mercury (Hg), lead (Pb), cadmium (Cd), and copper (Cu) in* Alpinia oxyphylla* Miq. Compared to the criteria of heavy metals, the contents of As, Hg, Pb, and Cd in almost all the samples were in accordance with The Green Trade Standards. The contents of Cu were higher than the criteria for heavy metals except the samples from Changxing town, Qiongzhong county, Maoyang town, Qiongzhong county, Wupo town, Tunchang county, and Nanlv town, Tunchang county, in Hainan province. The best cultivation regions of* Alpinia oxyphylla* Miq. were from Changxing town, Qiongzhong county, Maoyang town, Qiongzhong county, Wupo town, Tunchang county, and Nanlv town, Tunchang county, in Hainan province. This research would provide the scientific basis for quality control and standardization of* Alpinia oxyphylla* Miq.

## 1. Introduction

*Alpinia oxyphylla* Miq., known as Yizhi in Chinese, was a dry ripe fruit of herbaceous perennial belonging to Zingiberaceae, mainly produced in Hainan, Guangdong, Guangxi, and Yunan province [1]. It was one of the four big and sough medicines in China [2].

Modern pharmacological studies had shown that* Alpinia oxyphylla* Miq. had many pharmacological effects such as anti-inflammatory activities, antiallergy, antiulcer, and neuroprotective roles [3, 4], and it was commonly used in traditional East Asian medicine for the treatment of diarrhea, intestinal disorders, dieresis, frequent ruination, and urinary incontinence [5–7].

Chemical analysis revealed that* Alpinia oxyphylla* Miq. contained flavonoids, diarylheptanoids, sesquiterpenes, volatile oil, steroids, and their glycosides, and so forth [8]. Our lab had reported the content levels of pharmacologically active chemicals including flavonoids, diarylheptanoids, and sesquiterpenes, between the seeds and pericarps of* Alpinia oxyphylla *capsular fruit gathered from different production [5]; also, both individual and total contents of 10 nucleobases and nucleosides in the fruit of* Alpinia oxyphylla* Miq. collected from their famous regions in southern China were reported [9]. The results indicated that there was a large variation in the contents of pharmacologically active chemicals among the herbs from different regions.

While many investigations on the quality values of* Alpinia oxyphylla* Miq. were being reported, less emphasis had been made on the heavy metals. Besides, concentrations of heavy metals present in medicinal plants were, aside from other organic plant compounds, of great importance in order to understand their pharmacological actions [10].

The present study was conducted to establish baseline information for heavy metals in* Alpinia oxyphylla* Miq. The results are compared to the limitation of heavy metals in The Green Trade Standards issued by the State Department of Commerce in 2001 [11]. 20 batches of* Alpinia oxyphylla* Miq. gathered from their famous regions in southern China were selected. The contents of arsenic (As), mercury (Hg), lead (Pb), and cadmium (Cd) were determined by atomic fluorescence spectroscopy (AFS), and the contents of copper (Cu) were determined by atomic absorption spectroscopy (AAS). The information gained may help upcoming research to focus on the site of nutrition, pharmacology, and toxicology.

## 2. Experimental

### 2.1. Apparatus

For measurements, 3510 Atomic Absorption Spectrophotometry (Agilent Technologies Instrument Co., Ltd., Shanghai, China) equipped with Cu hollow cathode lamps (Beijing Titan Instruments Co., Ltd., Beijing, China) was used. A model AFS 830 sequential injection vapor generation double-channel nondispersive atomic fluorescence spectrometry (Beijing Titan Instruments Co., Ltd., Beijing, China) equipped with As, Hg, Pb, and Cd hollow cathode lamps (Beijing Titan Instruments Co., Ltd., Beijing, China) was used. A LabTech EG20A electric hot plate (Beijing Titan Instruments Co., Ltd., Beijing, China) was employed for sample preparation.

### 2.2. Regents

Chemicals and reagents used throughout this work were of suprapure grade. Ultrapure water was used throughout the experiment. Certified stock standards (1000 mg·L^−1^, each) for As, Hg, Pb, Cd, and Cu were purchased from the National Research Center for Certified Reference Materials (NRCCRM, China). Dilutions of standards and samples were done using deionized water (18.2 MΩ cm). These reagents, such as hydrochloric acid, sulfuric acid, dithizone, carbon tetrachloride, potassium hydroxide, cobalt chloride, thiourea, oxalic acid, potassium ferricyanide, ascorbic acid, and lanthanum nitrate, were purchased from Guangzhou chemical reagent factory. Potassium borohydride was purchased from Sinopharm Chemical Reagent Co., Ltd.

### 2.3. Sample Collection

Twenty samples of* Alpinia oxyphylla* Miq. were collected from different cultivation regions such as Hainan, Guangdong, Guangxi, and Yunnan province of China, as given in Table 1. After collection, the samples were dried at 45°C for 6 days. Botanical identification and authentication of the collected species with depositions of herbarium specimens had been done by Jianping Tian, associate professor of Hainan medical university. The voucher specimens were deposited at the Herbarium of Hainan Medical University.

### 2.4. Sample Preparation

Approximately 1.000 g of the powdered samples was weighed and transferred into 100 mL conical flask and mixed with 10 mL HNO_3_ and 2 mL HClO_4_. After addition of all digestion reagents, the samples were soaked overnight. Then, the flask was placed on the electric hot plate for digesting until the white smoke appeared. However, this operation had to be handled gently, ensuring that the digestion solution cannot be heated to dryness. After cooling, the digested solution was transferred to a 25 mL volumetric flask, and 5 mL of 100 g·L^−1^ thiourea solution was mixed and then diluted to 25 mL with 1.5% HCl. This solution was set aside for 30 min at room temperature for the determination of As, Hg, and Cu [12].

1.000 g of the powdered samples was weighed and prepared according to the same method for preparing for the solution of As. Then, the samples were digested until the digestion solution was heated to dryness. After cooling, the rest of the material was dissolved by 1.5% HNO_3_. The digesting solution was transferred to a 25 mL volumetric flask and then diluted to 25 mL with 1.5% HNO_3_. This solution was set aside for 30 min at room temperature for the determination of Pb.

1.000 g of the powdered samples were weighed and prepared and digested according to the same method for preparing for the solution of Pb. After cooling, the rest of the material was dissolved by 0.2 mol·L^−1^ H_2_SO_4_. The digesting solution was transferred to a 25 mL volumetric flask, and 5 mL of 0.5 g·L^−1^ dithizone carbon tetrachloride solution was mixed. After shocking fiercely for 2 min, 10 mL of 50 g·L^−1^ sulfuric acid solution and 0.5 mL of 0.10 *μ*g·L^−1^ cobalt chloride solution were mixed and then diluted to 25 mL with 0.2 mol·L^−1^ H_2_SO_4_. This solution was set aside for 30 min at room temperature for the determination of Cd.

Blanks solutions were prepared in a similar way, achieving the same final concentrations.

1 mL of certified stock standards (1000 mg·L^−1^, each) of As, Hg, Pb, and Cd was mixed and then diluted to 1.000 mg·L^−1^ with 3% HNO_3_ as calibration solution.

### 2.5. Atomic Fluorescence Spectroscopy (AFS) Measurement

20 g·L^−1^ potassium borohydride solution was used as reductant and 5% HCl was used as carrier liquid for the determination of As. 20 g·L^−1^ potassium borohydride solution was used as reductant and 2% HNO_3_ was used as carrier liquid for the determination of Hg. 30 g·L^−1^ potassium borohydride solution and 100 g·L^−1^ potassium ferricyanide solution were used as reductant and 1.6% HCl was used as carrier liquid for the determination of Pb. 30 g·L^−1^ potassium borohydride was used as reductant and 0.20 mol·L^−1^ H_2_SO_4_ was used as carrier liquid for the determination of Cd.

### 2.6. Atomic Absorption Spectroscopy (AAS) Measurement

The measured spectral element lines were Cu 324.7 nm. The current of Cu hollow cathode lamps was 2 mA. Slit was 0.2 nm. The ratio of combustion air to acetylene was 8 : 1.5.

### 2.7. Sample Analysis

Calibration solutions of each element were prepared according to Table 2. The content of Cu was measured by AAS, and the contents of other four elements, that is, As, Hg, Pb, and Cd, were determined by AFS. The contents of each element of the samples were calculated by the standard curve.

Each sample was analyzed 3 times for the same condition. Relative standard deviation (RSD) of the data was less than 2%.

## 3. Results and Discussion

### 3.1. Methodological Validation

#### 3.1.1. Calibration Curve

The calibration curves, correlation coefficients, and linearity ranges of As, Hg, Pb, Cd, and Cu were listed in Table 3.

The calibration curves were linear up to 50 *μ*g·L^−1^ for As, 2 *μ*g·L^−1^ for Hg, 8 *μ*g·L^−1^ for Pb and Cd, and 2 mg·L^−1^ for Cu. The linear correlation was between 0.9985 and 0.9999, and the favorable correlation showed evidence for the reliability of the proposed method.

### 3.2. Accuracy and Repeatability Tests

The accuracy of the method was also evaluated by recovery experiments carried out using samples with known concentrations of As, Hg, Pb, Cd, and Cu and analyzed 6 times according to the test method. The RSD of 6 parallel tests was between 0.0% and 2.7% (Table 4).

Approximately 1.000 g of the powdered samples was weighed and prepared and analyzed for 6 times according to the test method. The RSD of repeatability tests was shown in Table 4. The overall variations were no more than 3.9%, indicating a good repeatability.

### 3.3. Recovery Tests

0.5000 g of the powdered samples was weighed and mixed with different calibration solution (80%, 100%, and 120% of the known amounts) of each element, then prepared, and analyzed for 3 parallel tests according to the test method. The results of recovery tests were shown in Table 5. The recoveries of each element ranged from 93.4% to 104.2%, with the RSDs < 2.6%.

### 3.4. The Contents of Heavy Metals of the Investigated Samples from Different Cultivation Regions

The contents of As, Hg, Pb, Cd, and Cu in 20 samples from different cultivation regions were analyzed by the standard curve method and the results were shown in Table 6.

“Green Trade Standards of Importing & Exporting Medicinal plants Preparations,” which was awarded by the State Department of Commerce of China in 2001, had set bounds to the content of heavy metals in medicinal plants. That is, the content of As, Hg, Pb, Cd, and Cu in medicinal plants is not more than 2.0, 0.2, 5.0, 0.3, and 20.0 mg·kg^−1^, respectively [12].

Compared to the criteria of heavy metals, the contents of As, Hg, Pb, and Cd in almost all the samples were in accordance with The Green Trade Standards, but the amounts varied with place remarkably. As seen in Table 6, the contents of As and Hg varied more significantly between the samples collected from Hainan province than that from Guangdong, Guangxi, and Yunnan province. It was lower in sample S7 (from Baisha, Hainan), S9 (from Changxing, Hainan), and S18 (from Xingxing, Hainan). The contents of Pb and Cd in sample S1 (from Yangjiang, Guangdong) reached the highest whereas it is lowest in sample S4 (Xishuangbannan, Yunnan) and S12 (from Limushan, Hainan).

The contents of Cu varied more significantly in the investigated samples. The content of Cu in sample S3 (from Nanning, Guangxi) accumulates as high as 155.5 mg·kg^−1^, whereas it is only 7.530 mg·kg^−1^ in sample S20 (from Nanlv, Hainan). The contents of Cu were higher than the criteria for heavy metals expect samples S10 (from Changxing, Hainan), S13 (from Maoyang, Hainan), S19 (from Wupo, Hainan), and S20 (from Nanlv, Hainan).

It was concluded from the results that the contents of As, Hg, Pb, Cd, and Cu in the samples collected from Hainan province, especially from Changxing town and Maoyang town in Qiongzhong county, and Wupo town and Nanlv town in Tunchang county, were lower than the criteria for heavy metals. These areas were the best cultivation regions for the security of heavy metals in* Alpinia oxyphylla* Miq.

## 4. Conclusion

20 batches of* Alpinia oxyphylla* Miq. were collected from Yunnan, Guangdong, Guangxi, and Hainan province in China. The contents of heavy metals of As, Hg, Pb, Cd, and Cu were determined and compared. The contents of Cu were determined by atomic absorption spectroscopy, and the contents of As, Hg, Pb, and Cd were determined by atomic fluorescence spectroscopy.

Compared to the criteria of heavy metals, the contents of As, Hg, Pb, and Cd in almost all the samples were in accordance with The Green Trade Standards, but the amounts varied with place remarkably. The contents of Cu were higher than the criteria for heavy metals expect samples S10 (from Changxing, Hainan), S13 (from Maoyang, Hainan), S19 (from Wupo, Hainan), and S20 (from Nanlv, Hainan).

The results indicated that geographical source might be a major factor to influence the contents of heavy metals of As, Hg, Pb, Cd, and Cu in* Alpinia oxyphylla* Miq. The best cultivation regions of* Alpinia oxyphylla* Miq. were from Changxing town, Qiongzhong county, Maoyang town, Qiongzhong county, Wupo town, Tunchang county, and Nanlv town, Tunchang county, in Hainan province.

This research had established the test method of heavy metals in* Alpinia oxyphylla* Miq. which would be helpful for monitoring heavy metals in the growth stage of* Alpinia oxyphylla* Miq. Besides, it would have some reference for the analysis of heavy metals in other herbs.

This research would provide the scientific basis for quality control and standardization of* Alpinia oxyphylla* Miq.

## Figures and Tables

**Table 1:** The samples of *Alpinia oxyphylla* Miq. from different cultivation regions.

Samples	Cultivation regions	Collecting time
S1	Yangjiang county, Guangdong province	13 July
S2	Daba town, Yangdong county, Guangdong province	4 July
S3	Nanning Medicinal Herb Botanical Garden, Guangxi province	14 June
S4	Xishuangbanna Tropical Botanical Garden, Yunnan province	18 May
S5	Yacha town, Baisha county, Hainan province	2 June
S6	Yacha town, Baisha county, Hainan province	23 June
S7	Yinggeling town, Baisha county, Hainan province	27 May
S8	Wushi town, Qiongzhong county, Hainan province	26 May
S9	Changxing town, Qiongzhong county, Hainan province	26 May
S10	Changxing town, Qiongzhong county, Hainan province	23 June
S11	Changxing town, Qiongzhong county, Hainan province	25 July
S12	Limushang town, Qiongzhong county, Hainan province	30 May
S13	Maoyang town, Qiongzhong county, Hainan province	11 May
S14	Xinglong town, Wanning county, Hainan province	12 May
S15	Sangengluo town, Wanning county, Hainan province	29 May
S16	Changfeng town, Wanning county, Hainan province	29 May
S17	Nanlin town, Wanning county, Hainan province	29 May
S18	Xingxing town, Tunchang county, Hainan province	3 June
S19	Wupo town, Tunchang county, Hainan province	21 July
S20	Nanlv town, Tunchang county, Hainan province	7 August

**Table 2:** The preparation of calibration solutions of heavy metals.

Element	1	2	3	4	5	6
As	0	2	5	10	20	50
Hg	0	0.1	0.2	0.5	1.0	2.0
Pb	0	5	10	20	40	80
Cd	0	0.5	1	2	4	8
Cu	0	0.2	0.5	1.0	1.5	2.0

**Table 3:** The calibration curves and correlation coefficients of each element.

Element	The standard curve	Correlation coefficient	Linearity range
As	*I* = 172.9*C* + 40.29	*r* = 0.9999	0~50 *μ*g·L^−1^
Hg	*I* = 857.1*C* + 20.58	*r* = 0.9997	0~2 *μ*g·L^−1^
Pb	*I* = 1482*C* + 213.9	*r* = 0.9985	0~8 *μ*g·L^−1^
Cd	*I* = 1240*C* − 162.0	*r* = 0.9991	0~8 *μ*g·L^−1^
Cu	*A* = 0.001438 + 0.136546*C*	*r* = 0.9998	0~2 mg·L^−1^

**Table 4:** The results of the accuracy of the method (*n* = 6).

Element	Precision (RSD%, *n* = 6)	Reproducibility (RSD%, *n* = 6)
As	1.7	3.9
Hg	2.7	1.7
Pb	1.4	0.72
Cd	2.1	3.9
Cu	0.0	2.2

**Table 5:** The results of recovery tests (*n* = 9).

Element	Content of sample (*μ*g.g^−1^)	Mass of added certified element (*μ*g·g^−1^)	Content by analyzed (*μ*g·g^−1^)	Recoveries (%)	Average recoveries (%)	RSD (%)
As	0.3754	0.4500	0.7824	90.5	93.4	2.6
		0.5500	0.9265	108.8		
		0.6600	1.0134	92.9		

Hg	0.0409	0.0180	0.0626	94.4	102.8	1.8
		0.0230	0.0677	101.3		
		0.0270	0.0718	112.8		

Pb	0.3791	0.3000	0.7086	109.8	101.9	1.3
		0.3800	0.7643	101.4		
		0.4600	0.8132	94.4		

Cd	0.0379	0.0120	0.0514	112.8	104.2	2.2
		0.0150	0.0519	93.5		
		0.0190	0.0582	106.3		

Cu	21.50	3.000	24.75	108.3	101.6	2.1
		4.000	24.13	93.8		
		5.000	26.63	102.5		

**Table 6:** The contents of As, Hg, Pb, Cd, and Cu of the investigated samples.

	As (mg·kg^−1^)	Hg (mg·kg^−1^)	Pb (mg·kg^−1^)	Cd (mg·kg^−1^)	Cu (mg·kg^−1^)
S1	0.0067	0.0095	0.0278	0.1405	27.50
S2	0.0115	0.0046	0.1214	0.0724	29.25
S3	0.0008	0.0230	0.0302	0.0640	155.5
S4	0.0650	0.0001	0.0092	0.0076	51.85
S5	0.0404	0.0092	0.0344	0.0181	67.50
S6	0.1025	0.0123	0.0702	0.0423	69.25
S7	0.0000	0.0051	0.0358	0.1244	23.75
S8	0.0000	0.0232	0.0117	0.0102	66.75
S9	0.0034	0.0061	0.0253	0.0414	40.75
S10	0.0678	0.0071	0.0818	0.0093	6.080
S11	0.0634	0.0080	0.0801	0.0095	8.130
S12	0.0190	0.0089	0.0437	0.0013	49.65
S13	0.0910	0.0053	0.0697	0.0086	18.03
S14	0.0495	0.0024	0.0991	0.0282	37.53
S15	0.0284	0.0022	0.0200	0.0165	99.75
S16	0.0053	0.0089	0.0253	0.0201	69.25
S17	0.1888	0.0073	0.0554	0.0132	99.00
S18	0.0076	0.0052	0.0564	0.0358	24.75
S19	0.0652	0.0226	0.1662	0.0166	7.830
S20	0.1055	0.0260	0.0599	0.0081	7.530
